# Attachment as a framework to facilitate empowerment for people with severe mental illness

**DOI:** 10.1111/papt.12316

**Published:** 2020-10-30

**Authors:** Cathelijn D. Tjaden, Cornelis L. Mulder, Philippe A.E.G. Delespaul, Arnoud R. Arntz, Hans Kroon

**Affiliations:** ^1^ Department of Reintegration and Community Care Trimbos Institute Utrecht The Netherlands; ^2^ Department of Social and Behavioral Sciences Tranzo Scientific Center for Care and Welfare Tilburg University The Netherlands; ^3^ Department of Psychiatry Erasmus Medical Center Rotterdam The Netherlands; ^4^ Antes Parnassia Psychiatric Institute Rotterdam The Netherlands; ^5^ School of Mental Health and NeuroSciences Maastricht University The Netherlands; ^6^ Mondriaan Mental Health Trust Maastricht/Heerlen The Netherlands; ^7^ Department of Clinical Psychology University of Amsterdam The Netherlands

**Keywords:** attachment, empowerment, interpersonal relationships, recovery, severe mental illness

## Abstract

**Objectives:**

Recovery and empowerment have evolved into key objectives in the treatment and care of people with severe mental illness (SMI), and interest has grown in the role of social relationships in recovery. This study is the first to explore whether attachment styles are related to levels of empowerment, and secondly, whether attachment anxiety and attachment avoidance are associated with lower empowerment levels, independently of quality and frequency of social contact.

**Design:**

We used a cross‐sectional design.

**Methods:**

In a sample of 157 participants with SMI in outpatient care, associations between attachment (Revised Adult Attachment Scale), self‐reported social functioning, and empowerment (Netherlands Empowerment List) were assessed.

**Results:**

Attachment anxiety and attachment avoidance were both associated with lower levels of empowerment. A stepwise multiple regression analysis showed that the prediction of empowerment was significantly improved by adding attachment anxiety and attachment avoidance to quality and frequency of social contact. Attachment anxiety, attachment avoidance, and quality of social contact were significant predictors; frequency of social contact was not.

**Conclusions:**

Although our design does not allow causal conclusions, our results highlight the importance of interpersonal processes and behaviours as routes to improving empowerment for people with SMI. A promising approach might thus consist of securing attachment bonds with significant others so that the self and the other are perceived as reliable resources. Our findings also feature the importance of reciprocity and equality in social relationships. Taken together, our study emphasizes the value of social, contextualized interventions in recovery work for people with SMI.

**Practitioner points:**

- Working towards attachment safety in interpersonal relations may be important in recovery‐oriented treatment and care for people with severe mental illness (SMI).
- Helping people with SMI to recognize and change how they tend to relate themselves to others may promote engagement and effectiveness of recovery‐oriented treatment and care.
- Reciprocity and equality in social relationships as vital complements to the more one‐sided nature of ‘standing alongside’ and offering support may be important requisites for empowerment.

## Background

Traditionally, severe mental illnesses (SMI) were considered chronic diseases with relapsing or deteriorating symptoms and poor prognoses (Bellack, 2005). Recovery was perceived as a medical outcome defined by remission of mental health symptoms and return to normality (Soundy et al., 2015). However, the consumer movement has stimulated a focus on a broadened definition of recovery within the mental health services (Slade, 2009). Here, recovery is conceptualized as ‘a personal, unique process of changing one's attitudes, values, feelings, goals, skills, and/or roles’, and ‘a way of living a satisfying, hopeful, and contributing life even within the limitations caused by illness’ (Anthony, 1993). The evidence for recovery practices and outcomes is increasing rapidly (Leamy, Bird, Le Boutillier, Williams, & Slade, 2011; Slade, 2009), and the enhancement of recovery is evolving into a key aspect of international mental health policies (see Slade et al., 2014). Recovery orientation in mental health care is now a fundamental principle of the World Health Organization’s Comprehensive Action Plan for Mental Health (WHO, 2013).

Empowerment is a key aspect of recovery from SMI (Davidson, O'Connell, Tondora, Lawless, & Evans, 2005; Leamy et al., 2011). It refers to a learning process focused on restoring a sense of self‐determination in everyday life by improving individuals’ levels of choice, influence, and control (Perkins & Zimmerman, 1995; Rappaport, 1981). The mental healthcare culture is moving towards more equitable and collaborative approaches with the ethic of empowering patients to make informed decisions (Anderson & Funnell, 2005; Barr et al., 2015). Empowered mental health consumers have a good self‐esteem, use health services more effectively, have improved abilities to manage their disease, and adopt healthier behaviour (Aujoulat, d’Hoore, & Deccache, 2007; Halvorsen et al., 2020; Linhorst & Eckert, 2003; Linhorst, Hamilton, Young, & Eckert, 2002; Wallerstein, 2006). Moreover, they believe to be self‐efficacious and are optimistic about the future (Corrigan et al., 1999a). Importantly, as well as an individual focus, empowerment entails a group dimension focused on the social and relational context of the process (Cattaneo & Chapman, 2010; Mezzina et al., 2006; Tew et al., 2012; Topor, Borg, Di Girolamo, & Davidson, 2011). Indeed, according to its working definition, empowerment does not occur in the individual alone, but includes a sense of connectedness with other people (Chamberlin, 1997; Leamy et al., 2011).

As a result, there is an increased interest in social relationships as a way to empower people within their own environment. In fact, social relationships and interactions have been identified as key agents of change in recovery (Corrigan & Phelan, 2004; Priebe, Burns, & Craig, 2013; Schön, Denhov, & Topor, 2009) and it has been found that at least one relationship that provides hope and encouragement is a critical factor in the process of recovery (Spaniol, Wewiorski, Gagne, & Anthony, 2002). This highlights the important task of mental health services to facilitate patient’s connectedness with others in a way that contributes to a social environment within which recovery and empowerment processes can take place.

However, people with SMI often experience difficulty in developing and maintaining social relationships (Davidson, Borg, et al., 2005; Whitley & Drake, 2010). Over half of them report feeling lonely (Perese & Wolf, 2005), they have fewer close relationships (Koenders, de Mooij, Dekker, & Kikkert, 2017) and not all relationships and social interactions are experienced as positive or supportive (Boydell, McKenzie, Van Os, & Murray, 2002; Tew et al., 2012; Yanos, Rosenfield, & Horwitz, 2001). Moreover, the emotional atmosphere within social relationships is found to be important: the risk of relapse can be greatly increased by a high level of expressed emotion (defined as intrusive over‐involvement or consistent patterns of criticism and hostility; Hooley, 2007). So although it is increasingly recognized that social factors are important to the process of empowerment, it remains unclear how individuals with SMI and their significant others can be supported in changing the characteristics of their relationship such that their interactions offer opportunities for support, engagement, and empowerment.

Attachment theory might provide a promising theoretical framework to enhance understanding in how to create such empowering interactions and support the development of positive relationships. Attachment theory proposes that one’s interpersonal relating styles emerge from early experiences with primary caregivers. As a child ages, internal working models about the self and others are developed, representing internalized beliefs and expectations in relationships. These models characterize attachment styles, and guide emotions, motives, and goals in interpersonal situations (Ainsworth & Bell, 1970; Bowlby, 1958, 1969). Attachment styles are assumed to be stable over time but recent research shows that they can change, according to context and recent experiences (Gillath & Karantzas, 2019; Kinley & Reyno, 2013; Levy et al., 2006; Mikulincer & Shaver, 2007a, 2007b).

Attachment is conceptualized in terms of two independent dimensions that underlie internal working models: attachment anxiety and attachment avoidance (Brennan, Clark, & Shaver, 1998). The dimension of attachment anxiety is also referred to as the model of self and is associated with a negative self‐perception and an excessive need to be approved by others. Attachment avoidance is referred to as the model of the other, and reflects the extent to which a person distrusts the goodwill of other people, and strives to maintain behavioural independence and emotional distance (Bartholomew & Horowitz, 1991; Shaver & Mikulincer, 2002).

**Figure 1:**
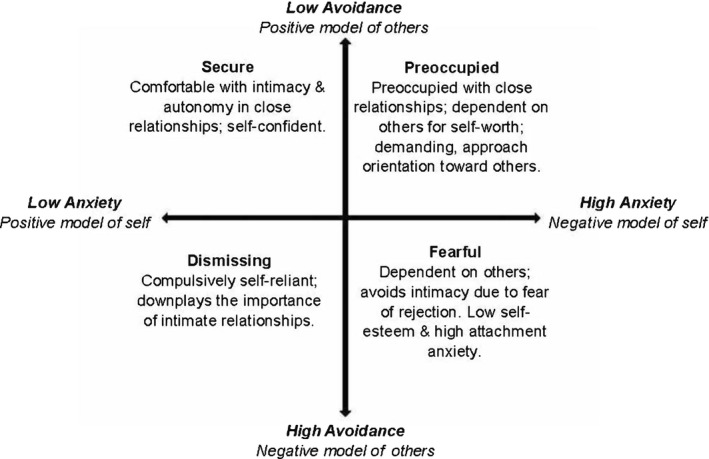
Bartholomew’s two dimensional, four‐prototype model of adult attachment.

An individual’s location at the intersection of these two dimensions yields four attachment prototypes, see Figure 1 (Bartholomew & Horowitz, 1991). Prototypically secure individuals score low on both dimensions. They have positive images of the self as deserving love and support, and perceive the other as a source of comfort and assistance. In contrast, individuals with an insecure attachment style have high levels in one or both dimensions: they are preoccupied (high anxiety, low avoidance); dismissing‐avoidant (low anxiety, high avoidance); or fearful‐avoidant (high anxiety, high avoidance; Bartholomew & Horowitz, 1991; Berry, Barrowclough, & Wearden, 2007). Since Bowlby’s influential work, a growing body of research has linked attachment insecurity to different forms of psychopathology (Alonso, Fernández, Fontanil, Ezama, & Gimeno, 2018; Crawford et al., 2007; Dagan, Facompré, & Bernard, 2018; Manning, Dickson, Palmier‐Claus, Cunliffe, & Taylor, 2017), including serious psychiatric disorders (Berry, Barrowclough, & Wearden, 2008; Bucci, Emsley, & Berry, 2017; Dozier, Lomax, Tyrrell, & Lee, 2001; Harder, 2014). However, even though research has linked attachment style with clinical outcomes, there has been little exploration of the potential link between attachment style and indicators of recovery.

Therefore, the present study intends to explore whether attachment theory can enhance our understanding of how to create social interactions within which recovery can take place by investigating the associations between attachment patterns and empowerment for people with SMI. Because attachment patterns shape individuals’ beliefs about their environment through a sense of self and others, they might support shaping beneficial environments in which people with SMI feel empowered. Greater understanding of attachment processes can then be useful for patients, family, friends, and practitioners in facilitating recovery. We hypothesized that (1) prototypical insecure attachment styles are associated with lower levels of empowerment and that (2) the dimensions of attachment anxiety and attachment avoidance are associated with lower levels of empowerment, independently of quality and frequency of social contact.

## Methods

### Study design

This study has a cross‐sectional design and is based on baseline data from a randomized controlled trial assessing the effectiveness of Resource Groups in Flexible Assertive Community Treatment (FACT) for people with SMI in the Netherlands. Details of the protocol are described elsewhere (Tjaden et al., 2019). FACT is the most widely used long‐term outpatient care for people with SMI in the Netherlands.

### Procedures

Patients were recruited between September 2017 and February 2019 at nine mental health centres throughout the Netherlands. The study population consisted of patients aged between 18 and 65 who met the Dutch consensus criteria for SMI (Delespaul & van Weeghel, 2013) and were expected to be treated by the FACT team for more than 12 months. Patients entering a FACT team (i.e. during intake) and those who had already been treated by the FACT team for no more than 24 months were eligible. Patients were excluded if they were unable to understand Dutch and/or to sign for informed consent. Care providers in the FACT team informed eligible patients on the study and invited them for participation. An independent researcher checked the in‐ and exclusion criteria and scheduled an appointment for signing informed consent and a face‐to‐face assessment that lasted approximately 90 min. Participants received a gift voucher worth €15. Socio‐demographic characteristics gathered during the interview included gender, age, marital status, education, employment status, and history of mental health and hospitalization.

### Material

#### Empowerment

The *Netherlands Empowerment List *(NEL; Boevink, Kroon, Delespaul, & Van Os, 2016) is a 40‐item self‐report questionnaire for measuring empowerment. Items were generated from a narrative, qualitative analysis of the recovery process in people with SMI. The NEL contains six subscales: ‘social support’ (seven items); ‘professional help’ (four items); ‘connectedness’ (six items); ‘confidence and purpose’ (12 items); ‘self‐management’ (five items); and ‘caring community’ (six items). A sample item from the ‘confidence and purpose’ scale is ‘I decide how I control my life’. Respondents rate their agreement on a 5‐point Likert‐scale ranging from 1 (*strongly disagree*) to 5 (*strongly agree*). The NEL displayed good internal consistency, moderate convergent validity, and good discriminant validity (Boevink et al., 2016). For this study, the mean of the total score was used (α = .92).

#### Attachment

The Revised Adult Attachment Scale (RAAS; Collins, 1996; Van Aken, van Bussel, & Wierdsma, 2017) is a 18‐item self‐report questionnaire intended to assess difficulties in adult attachment. The respondents answer items such as ‘I often worry that other people don't really love me’, on a 5‐point Likert‐scale ranging from 1 (*not at all characteristic of me*) to 5 (*very characteristic of me*). The scale consists of three subscales, each containing six items: ‘close’, ‘depend’, and ‘anxiety’ (Collins, 1996). The items of the ‘close’ and ‘depend’ subscales were reverse scored and averaged to form an overall index of the ‘attachment avoidance’ dimension (12 items) that reflects the degree to which individuals tend to avoid intimacy and interdependence with others (α = .78). The ‘anxiety’ subscale comprises an index of the ‘attachment anxiety’ dimension (six items) that reflects the degree to which a person is worried about being rejected or unloved (α = .84). Participants responded in terms of their general orientation towards close relationships (Collins & Feeney, 2000; Collins, Ford, Guichard, & Allard, 2006). The reliability of the RAAS is satisfactory to good (Collins, 1996; Collins & Feeney, 2004; Tait & Birchwood, 2004).

The two dimensions generate four prototypical attachment styles: secure, dismissive, preoccupied, and fearful. To this end, we z‐transformed the scores so that the two dimensions cross at zero and the standard deviation equalizes the spread. See Collins and Feeney (2004) for this procedure. While categorical representations are often used in a clinical setting, dimensional representations are preferred for research purposes (Fraley & Shaver, 2000). In this study, we used both representations of attachment in order to both appeal to a wide, clinical audience and obtain a deeper comprehension of the results. Figure 1, presented by Allison, Bartholomew, Mayseless, and Dutton (2008), shows the features and characteristics of the dimensional and categorical representations of attachment.

#### Frequency and quality of social contact

To obtain information on social functioning, subjects self‐reported the frequency and quality of social contacts over the past 3 months for five different categories: ‘family’, ‘friends’, ‘acquaintances’, ‘colleagues’, and ‘general’. Per category, the frequency of social contact was assessed on the basis of questions such as ‘In the past 3 months, how frequently did you see your friends?’. Answers were rated on a 7‐point scale ranging from 1 (*daily*) to 7 (*not at all*). The perceived quality of social contact per category was assessed on the basis of items such as ‘In the past 3 months, it was pleasant to see my friends’. Answers were rated on a 5‐point scale ranging from 1 (*always*) to 5 (*never*). If participants had indicated they had not seen their friends in the past 3 months, they did not fill in the questions on the quality of the contact. Participants who did not work did not fill in questions on contact with colleagues. Scores over the 5 groups were averaged to assess frequency (α = .63) and quality of social contact (α = .83).

### Data analysis

The data was stored using an online encrypted server (Jambo) and all analyses were performed using SPSS, version 25 (IBM). One participant quit the assessment after finishing under 10% of the questions; the data was removed. Before the hypotheses were tested, the following analyses were conducted. First, the data was checked, using boxplots for outliers and kurtosis and skweness *z*‐scores for normal distributions. Second, we computed frequency distributions, and mean and standard deviations for the subjects’ socio‐demographic characteristics, empowerment, attachment style, and the frequency and quality of social contact. Last, to explore associations, we determined correlations between empowerment, attachment dimensions (i.e. anxiety and avoidance), and measurements of social functioning (i.e. frequency and quality of social contact).

To test the first hypothesis – whether prototypical insecure attachment styles are associated with lower levels of empowerment – we performed an univariate analysis of variance (ANOVA), comparing intergroup differences in attachment styles on empowerment. We then converted the attachment styles into dummy variables, with secure attachment style as the reference category, and used a linear regression to predict the empowerment score. For the second hypothesis, we used the dimensional representation of attachment. A hierarchal regression analysis was performed to determine whether the two attachment dimensions predicted empowerment scores, independently of frequency and quality of social contact. To this end, the measurements of social functioning (frequency and quality of social contact) were entered into the model in the first step, and attachment anxiety and attachment avoidance were entered in the second step. The level of statistical significance for all analyses was set at *p* < .05.

## Results

### Sample characteristics

The definitive sample consisted of 157 participants aged 20–66 (*M* = 40.17 years, *SD* = 11.2), 93 (59%) male and 65 (41%) female. Thirty‐three per cent of the sample had a partner and 45.6% had children. Most had been born in the Netherlands (79%). The highest completed educational level varied: 4.4% of the participants had not finished any education, 19.1% had completed primary school, 58.3% had finished secondary school, and 17.1% had finished college/university. Half of the participants (50.1%) of the participants were unemployed, 13.9% were in paid employment and 15.2% did volunteer work. Mean self‐reported age at first contact with the mental health services was 28.3 years (*SD* = 12.7, range = 6–60), and mean self‐reported duration of contact with these services was 8.1 years (*SD* = 7.45, range = 0.08–35.00). Seventy‐three per cent of the sample had been hospitalized in their life, 23.3% of them more than three times.

### Attachment style and empowerment

**Figure 2:**
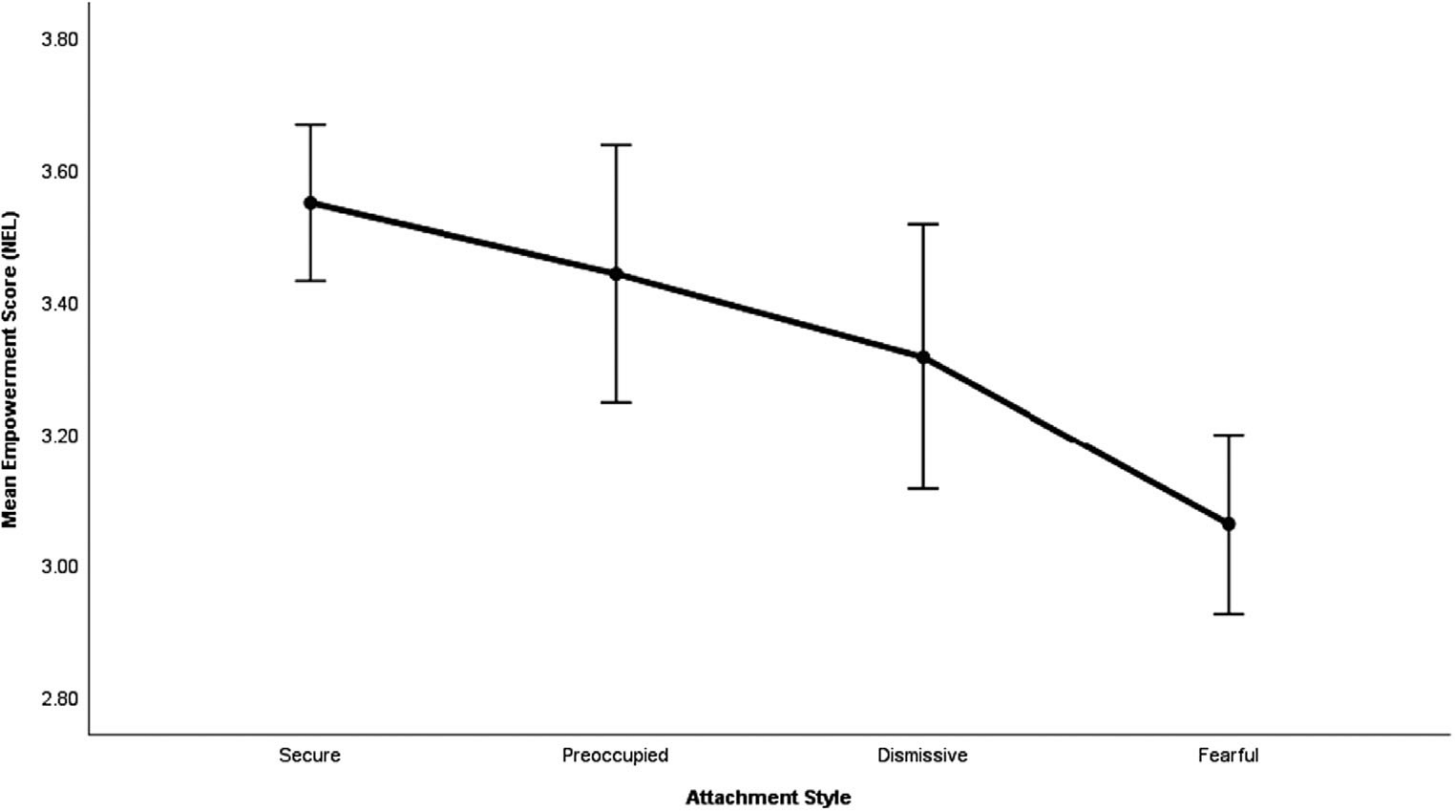
Mean empowerment score (NEL) for the four prototypical attachment styles. Error bars represent 95% CI.

We first explored the correlation of some demographics (age, gender, education) with the mean NEL total score. As none of these were significant, we did not include these in the further analyses. To test the first hypothesis we divided participants into one of the four categorical attachment styles (Collins & Feeney, 2004). This produced 52 (32.9%) patients with a secure attachment style, 28 (17.7%) with a preoccupied attachment style, 23 (14.6%) with a dismissive attachment style, and 54 (34.2%) with a fearful attachment style. The mean NEL total score differed significantly between attachment styles (*F*_3,153_ = 10.12, *p* < .001). More specifically, the dummy regression showed that the empowerment scores of patients with a secure attachment style were significantly different from those of patients with a dismissive attachment style (β = −.245 *p* < .05) and from those of patients with a fearful attachment style (β = −.500, *p* < .001). See Figure 2.

### Attachment dimensions, social functioning, and empowerment

To test whether attachment anxiety and attachment avoidance would predict empowerment scores independent of the social functioning measures, we first explored correlations between the variables (Table 1). This showed that attachment anxiety and attachment avoidance were moderately correlated (*r* = .5), indicating related but distinct aspects of the same construct. Attachment anxiety and avoidance were both significantly correlated with the mean NEL empowerment score. Quality of social contact was also significantly correlated with the mean NEL empowerment score, but frequency of social contact was not.

**Table 1:** Pearson correlation coefficients between the variables

Variable	1a	1b	1c	1d	1e	1f	1g	2	3	4a	4b	4c	4d	5	6
1 Empowerment															
1a Mean total score	1														
1b Confidence and purpose	.876**	1													
1c Social support	.726**	.446**	1												
1d Caring community	.665**	.435**	.472**	1											
1e Connected‐ ness	.788**	.604**	.553**	.405**	1										
1f Self‐management	.798**	.813**	.400**	.353**	.575**	1									
1g Prof help	.376**	.133	.277**	.247**	.313**	.160**	1								
2 Attachment anxiety	−.403**	−.312**	−.372**	−.356**	−.211**	−.330**	−.126	1							
3 Attachment avoidance	−.440**	−.348**	−.436**	−.271**	−.361**	−.276**	−.171	497***	1						
4 Attachment style^a^, ^b^															
4a Safe	.295**	.246**	.275**	.181	.200	.219**	.130	−.692**	−.633**	1					
4b Preoccupied	.098	.102	.087	.003	.128	.019	.063	.343**	−.291**	−.325**	1				
4c Dismissive	−.014	−.023	−.005	.119	−.087	.007	−.110	−.243**	.264**	−.289**	−.192	1			
4d Fearful	−.383**	−.328**	−.353**	−.291**	−.252**	−.253**	−.103	.590**	.666**	−.505**	−.334**	−.297**	1		
5 Frequency of social contact	.128	.109	.139	−.001	.254**	.027	.026	.043	−.086	−.032	.042	−.002	−.012	1	
6 Quality of social contact	.615***	.491**	518**	.313**	.558**	.458**	.293**	−273***	.384***	.182	.120	−.099	−.199	.219**	1

Note

^a^ Correlational values of the different attachment style (represented as dummy variables) and the other continuous variables are Point Biseral correlation coefficients.

^b^ Correlational values between the different attachment styles (represented as dummy variables) are Phi correlation coefficients.

*** *p* < .001.

** *p* < .01.

We next performed a hierarchal multiple linear regression to predict the mean empowerment score. Quality of social contact and frequency of social contact was entered in the first step, and attachment anxiety and avoidance were entered in the second step. As Table 2 shows, addition of the two dimensions of attachment significantly improved the prediction of empowerment. The final model explained 44.1% of the variance (*R* ^2^ = .455; Adjusted R^2^ = .441; *F*_4,151_ = 31.51, *p* < .001). Quality of social contact was a significant predictor of empowerment (β = .50, *p* < .001), and attachment anxiety and attachment avoidance were negative significant predictors (β = −.19, *p* < .01 and β = −.16, *p* < .05 resp.). In neither model was frequency of social contact a significant predictor.

**Table 2:** Regression model for predicting empowerment scores (NEL, outcome variable) from the frequency of social contact, the reported quality of social contact, and attachment (predictor variables)

	*b*	*SE b*	β
Step 1			
Constant	1.723	.197	
Frequency of social contact	−0.004	.030	−.009
Quality of social contact	0.433	.046	.617***
Step 2			
Constant	2.648	.287	
Frequency of social contact	0.005	.028	.011
Quality of social contact	0.352	.047	.501***
Attachment avoidance	−0.123	.058	−.155*
Attachment anxiety	−0.099	.037	−.189**

Note

*R*^2^ = .38 for Step 1; *R* ^2^ = .46 for Step 2 (*p*s < .001).

* *p <* .05.

** *p <* .01.

*** *p <* .001. *b* represents unstandardized regression weights, *SE b* the standard error for the unstandardized regression weights and *β* indicates the standardized regression weights.

## Discussion

To the best of our knowledge, this is the first study to examine the relevance of attachment theory to facilitating empowerment in people with SMI. Our findings suggest that the incidence of insecure attachment patterns is high in people with SMI. They also showed an association between insecure attachment patterns and decreased empowerment. As expected, when entered in a regression model with quality and frequency of social contact, the two attachment dimensions– attachment anxiety and attachment avoidance – were significant predictors for empowerment scores. This indicates not only that attachment problems are highly prevalent, but that they may obstruct recovery‐based social and societal interventions. To shape empowering social relationships, and to maximize engagement and the effectiveness of recovery‐oriented treatment and care, people with SMI may therefore benefit from insights from attachment‐oriented interventions.

Given the high predictive value of quality of contact to higher empowerment scores, our findings highlight the importance of creating social environments that facilitate empowerment processes. Rather than being a function of the frequency of social contacts and activities, such empowering environments seem to depend on their perceived quality. These findings are in line with a substantial body of research that argues for the need to include the social context in understanding, analysing, and responding to people’s mental health difficulties (Dixon et al., 2009; Tew et al., 2012; Topor et al., 2006, 2011). They also show the key importance of positive social interactions in contributing to recovery in people with SMI (Chou & Chronister, 2012; Corrigan & Phelan, 2004). Moreover, previous evidence suggests that rather than focusing on increasing the number of social contacts and relationships, social interventions should emphasize their quality (Davidson, Borg, et al., 2005; Webber & Fendt‐Newlin, 2017).

To build on this, our study sought to take a further step towards understanding how social relations are perceived as empowering by investigating attachment patterns. As with previous studies (Berry et al., 2007; Carr, Hardy, & Fornells‐Ambrojo, 2018; Korver‐Nieberg, Berry, Meijer, de Haan, & Ponizovsky, 2015), our results suggest that a majority of people with SMI have an insecure attachment style and are therefore prone to difficulties in trusting and relying on others and themselves. Notably, our results suggest that this influences the degree of empowerment. Indeed, the prediction of empowerment scores was improved when the two dimensions attachment anxiety (i.e. model of self) and attachment avoidance (i.e. model of other) were added to measurements of social functioning. In the final model, attachment anxiety, attachment avoidance, and quality of social contact were significant predictors, while frequency of social contact was not. With regard to empowerment, this indicates that attachment is a distinct and important component of satisfying social contact, not merely a function of it.

Our results suggest that low attachment anxiety – in other words, a person’s sense of self as capable, competent, and having something to offer in relation to significant others – is an important requisite for empowerment. This highlights the importance of reciprocity and equality in social relationships as a vital complement to the more one‐sided nature of ‘standing alongside’ and offering support (Tew et al., 2012). For as long as social contacts are characterized by the latter, the working models of the fragile, unlovable self and the strong, knowing other may be confirmed – thereby verifying the characterizing tendency in attachment anxiety to depend on others for personal validation, acceptance, and approval. As this might, in turn, stimulate feelings of being dependent on others, it would stand in the way of developing a sense of autonomy and agency that is essential for empowerment (Mancini, 2007; Nelson, Lord, & Ochocka, 2001). Hence, a degree of mutuality and equality within relationships is important to improving one’s sense of self‐worth (Tew, 2013; Wyder & Bland, 2014). This supports the view that a functional sense of self or identity is an important factor in recovery, and in facilitating effective coping and mobilization of support (Davidson & Strauss, 1992; Tait & Birchwood, 2004).

The negative predictive value of attachment avoidance on empowerment indicates that the process of empowerment is also interfered by a pattern in which a person downplays the importance of close relationships, has little confidence in others, and defensively denies the need for their support. If people do not trust others’ goodwill and strive to maintain emotional distance, they will be unable to build safe social relationships, thus discarding potential sources of support. This reinforces the notion that empowerment is not the same as being able to do everything independently, but involves actively choosing to let others in, ask for help, and develop trust in them (Davidson et al., 2008; Pernice‐Duca, 2010; Zimmerman, 1995). Indeed, a crucial part of recovery is choosing to move towards rather than away from others (Corrigan et al., 1999).

### Implications

In short, our results show that relational views of the self and others are substantial components in facilitating empowerment. This has several theoretical and clinical implications for working towards empowerment for people with SMI. Most importantly, rather than working with individual members, an attachment framework would emphasize the importance of increasing empowerment through a focus on relationships within social systems. Hence, by creating a secure base that facilitates connectedness within this system and exploration outside of it; treatment and care would focus on shifting the mutual attachment relationships within a social system towards greater security (Byng‐Hall, 2008). Hereby, working with attachment relations is a way of perpetuating the role of the interpersonal world in treatment and care. Both individual treatments (Levy et al., 2006) as well as family attachment interventions that target the family attachment system as a framing device (Liddle & Schwartz, 2002) describe different ways towards transforming impaired and distorted representations of self and others in order to create security within a social system. The development of bidirectional and supportive relationships is one aspect of such secure base. Moreover, working towards understanding the past from everyone’s perspective, expressions of forgiveness and acceptance, and open communication are all essential parts that constitute a secure base, change the mutual relational styles, and have the potential to modify internalized attachment representations (Byng‐Hall, 1999; Keiley, 2002; Liddle & Schwartz, 2002; Rutter & Sroufe, 2000; Shaw, Bell, & Gilliom, 2000).

In addition, the notion of epistemic trust might be important in the development of a secure base that is characterized by trustful mutual collaboration partnerships in order to facilitate empowerment. Epistemic trust describes the willingness to accept new information from another person as trustworthy, generalizable, and relevant and it allows individuals to benefit and learn from their (social) environment (Fonagy & Allison, 2014; Fonagy, Luyten, & Allison, 2015; Fonagy, Luyten, Allison, & Campbell, 2017). In other words, in order to be able to develop meaningful partnerships and to turn to others in time of need to make sense of what is happening to us, individuals need a workable level of epistemic trust. To facilitate empowerment by creating attachment safety in a social system, future studies could therefore consider the three communicational systems that are maintained to restore epistemic trust (see Fonagy & Allison, 2014). The notion of epistemic trust constitutes a shift towards a socially oriented perspective and to interventions that target both malignant and beneficial aspects of the environment (Fonagy et al., 2015), and it also emphasizes the importance of a good therapeutic relation. That is, the feeling of being understood, supported, and valued within the therapeutic relation is seen as an essential starting point which makes life outside treatment and care a setting in which new information about oneself and the other can be acquired and internalized (Fonagy & Allison, 2014; Fonagy & Campbell, 2017).

Taken together, we argue that the facilitation of the process of empowerment of the patient should be considered in the context of the interpersonal and social world so that relations with significant others, such as family, friends, and professionals, become meaningful working mechanisms in treatment and care. Importantly, a good therapeutic relation might be fundamental to engage readiness for patients to step into beneficial partnerships with their social environment. Future research can rely on these theoretical advances to further investigate how to establish a social environment that is characterized by safe attachment bonds in order to facilitate empowerment.

According to the social baseline theory (Coan, 2008), developed from the social neuroscience of attachment processes, the human brain evolved in a highly social environment. The presence of other people helps individuals to conserve important and metabolically costly resources. Therefore, rather than conceptualizing human beings as separate entities, it makes more sense to consider social relatedness and its mental correlates as the normal ‘baseline’ condition (Beckes & Coan, 2011; Coan, 2008; Mikulincer & Shaver, 2012). Using this as a starting point helps us to understand why experiences of separation, loneliness, rejection, abuse, and neglect are so detrimental and distressing, and why restoring functional and safe social relationships is so essential to recovery and empowerment.

### Limitations

The current findings have to be interpreted in light of the following limitations. First, our findings are based on cross‐sectional data, which limits causal conclusions on the influence of changes in attachment for empowerment. Given various promising attempts to revise and modify attachment during treatment (Gillath & Karantzas, 2019; Liddle & Schwartz, 2002; Stavrianopoulos, Faller, & Furrow, 2014; Travis, Bliwise, Binder, & Horne‐Moyer, 2001), we would recommend that future studies use longitudinal data to explore whether attachment patterns could indeed be a working mechanism for bringing about changes in empowerment. In addition, applying mediation analyses on longitudinal data would be helpful in order to further investigate whether quality of social contact is in fact a mediator between attachment and empowerment. Also, the effect sizes for attachment dimensions appear rather small, with quality of social support being much larger. This does not invalidate the role of attachment, but does suggest a nesting within a more complex set of factors. Longitudinal data and mediation analysis would be helpful to unravel the different factors that influence empowerment.

Second, psychotic episodes and levels of positive and negative symptoms have been argued to influence attachment styles (Korver‐Nieberg, Berry, Meijer, & de Haan, 2014); if the course of illness is more severe, an individual may develop more difficulties in attachment relationships and therefore a more insecure attachment style. For this reason, it is not fully understood whether attachment style is predictive of symptoms of illness, or whether it changes as a result of the illness (Carr et al., 2018; Korver‐Nieberg et al., 2014). Our patient sample had a range of diagnoses, including affective and non‐affective psychosis, bipolar disorder, and personality disorder. As we did not control for symptomatic levels of any kind, the influence of fluctuating symptoms on attachment scores cannot be ruled out, and require longitudinal studies. Related to this, we did not control for factors potentially influencing the association. Therefore, future studies should include other variables (i.e. depression, loneliness, and having a partner) or apply tighter inclusion/exclusion criteria to further isolate and clarify the effect.

Third, it could be argued that empowerment and attachment – which derive from two different fields of research and practice – are essentially two sides of the same coin, both involving situations and influences that make people feel that they are important and matter to themselves and the world around them. Indeed, we found high correlational values between (the subscales of) the constructs, as reported in Table 1. However, examinations of the items in the two questionnaires made us doubt their similarities. While the RAAS mainly concerns relational distance from and trust in others, the NEL clearly assesses a broader range of areas in life. Some questions concern significant others, support and feeling accepted, while others assess hope for the future, having purpose in life, insight into autobiographical events, and being able to do things that matter. Nevertheless, future research should further investigate the overlap and distinctness of the two constructs.

Lastly, we argue that a two‐dimensional method of assessing attachment should be used to include the perspectives of practitioners and significant others (i.e. involved family members and close friends). This would provide insight into the bilateralism of the attachment patterns and the subsequent approach to enhancing attachment safety of the social environment. Indeed, problematic relationship styles may reflect low self‐esteem on the part of carers (Kuipers et al., 2006), indicating that, if the relationship is to recover, carers may need support too (Tew et al., 2012). As strengths, resources, and vulnerabilities in the network become visible, insight into the interaction would facilitate system changes.

### Conclusions

Empowerment is increasingly recognized as an important objective in the treatment and care for people with SMI. Our main finding – that attachment is a consistent predictor of empowerment for people with SMI – is important in the context of its clinical applications, as it indicates the significance of interpersonal processes and behaviours for improving empowerment. We show that a majority of the people with SMI have insecure attachment patterns, and therefore find it difficult to trust and rely on others and themselves. This complicates social interventions and may explain the social difficulties and loneliness that people with SMI experience. In line with attachment theory, it might be important that those in a patient’s social environment all develop alternative coping strategies to adjust interpersonal attachment safety. It then follows that to achieve sustainable alterations in empowerment the focus of treatment should be broadened towards system changes. Hereby, our study emphasizes the value of social, contextualized interventions in recovery work for people with SMI.

## Conflicts of interest

All authors declare no conflict of interest.

## Author contributions

C.D. Tjaden, Ph.D (Conceptualization; Data curation; Formal analysis; Investigation; Methodology; Project administration; Resources; Visualization; Writing – original draft) Niels L. Mulder (Conceptualization; Formal analysis; Funding acquisition; Methodology; Supervision; Validation; Writing – original draft; Writing – review & editing) Philippe A.E.G. Delespaul (Conceptualization; Formal analysis; Validation; Writing – review & editing) Arnoud R. Arntz (Conceptualization; Validation; Writing – review & editing) Hans Kroon (Conceptualization; Data curation; Formal analysis; Funding acquisition; Methodology; Resources; Supervision; Validation; Writing – original draft; Writing – review & editing).

## Ethical approval

The protocol and consent procedures were approved before study initiation by the Medical Ethical Committee at VU Medical Centre (IDS: 2017.316). The local ethical Review Boards or Ethical Committees at the participating mental health centres also approved participation.

## Data Availability

The data that support the findings of this study are available in [Mendeley Data] at http://dx.doi.org/10.17632/wbkc8zghyf.1
